# Portal Venous Stenting in Locally Advanced Pancreatic Cancer to Decrease Risk of Thrombosis Before Irreversible Electroporation: A Case Report and Review of the Literature

**DOI:** 10.1089/pancan.2016.0022

**Published:** 2017-03-01

**Authors:** Justin F. Monroe, Thor Johnson, Barish H. Edil

**Affiliations:** ^1^ Department of Radiology, University of Colorado at Denver—Anschutz Medical Campus, Aurora, Colorado.; ^2^ Department of Surgery, University of Colorado at Denver—Anschutz Medical Campus, Aurora, Colorado.

**Keywords:** irreversible electroporation, locally advanced pancreatic cancer, portal vein, venous stenting, portal vein thrombosis

## Abstract

**Background:** For patients with locally advanced pancreatic cancer, irreversible electroporation (IRE) is a fairly novel treatment tool that has shown promise in improving survival. However, many patients being considered for IRE have tumors adjacent to and/or encasing portal vasculature, increasing risk of postoperative portal vein thrombosis and associated complications. This report describes a successful new approach of portal venous stenting preoperatively to decrease this risk.

**Case Presentation:** A 64-year-old female with locally advanced pancreatic cancer, initially deemed too high risk for IRE therapy because of portal vein–superior mesenteric vein confluence encasement and compression, was offered and underwent venous stenting to decrease the chance of postoperative thrombosis and related complications. Stenting improved portal venous flow, decreased collateralization, and allowed for successful IRE. At 61 days post-IRE, there was no significant tumor growth and the stent remained patent.

**Conclusion:** Preoperative portomesenteric stenting could expand the population eligible for IRE therapy, allowing for this treatment in patients without other options. To the authors' knowledge, this is the first reported case of portal venous stenting for this purpose.

## Introduction and Background

Pancreatic cancer is the second most common gastrointestinal malignancy, behind only to colorectal cancer. As most early pancreatic tumors are clinically silent, ∼80% of patients are not surgical candidates at diagnosis.^1^ Many of these patients are classified as stage III and then further categorized as locally advanced pancreatic cancer, defined as nonresectable because of local vascular involvement with or without local lymph node invasion. Chemotherapy and radiation have been the mainstays of treatment for this patient population. Recently, clinicians have begun to explore the use of irreversible electroporation (IRE) in this cohort, a nonthermal ablative therapy able to destroy tumor cells while leaving vital adjacent vessels unharmed. IRE has shown promise in improving survival.^2^ However, there are reports of post-IRE portal vein thrombosis (thought to be related to increased compression from postoperative edema), even resulting in death in some circumstances.^3–5^ This report describes a new approach of portal venous stenting preoperatively to decrease this risk.

## Presentation of Case

**FIG. 1.**
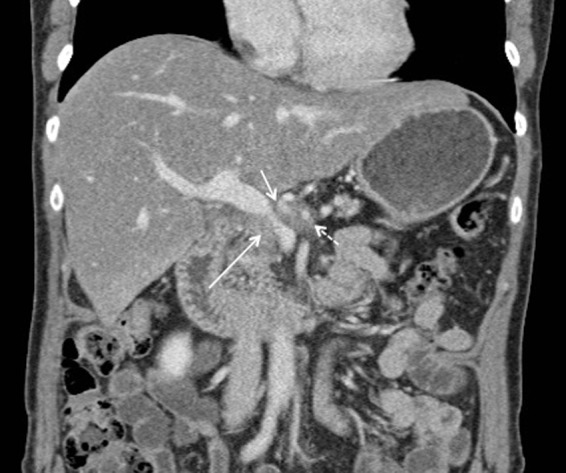
Coronal contrast-enhanced CT showing portal vein–SMV confluence narrowing because of extrinsic tumor compression (long solid arrow). Also seen is proximal common hepatic artery (short solid arrow) and proximal splenic artery (short dashed arrow) encasement, without SMA involvement. SMV, superior mesenteric vein.

A 64-year-old Caucasian female with locally advanced pancreatic head adenocarcinoma was being followed in surgical oncology clinic for possible further treatment options. Owing to tumor compression/encasement of the portal vein–superior mesenteric vein (SMV) confluence, proximal common hepatic artery, and proximal splenic artery, she was not a surgical candidate for resection and had already undergone both chemotherapy and stereotactic body radiation therapy (SBRT) with stable disease burden for 6 months. IRE was being considered; however, given this patient's significantly narrowed portal vein–SMV confluence (Fig. 1), the procedure was deemed too high risk for fear of postoperative portal vein thrombosis. To reduce this risk, we proposed stenting the portal–SMV confluence before open surgical exploration and expected IRE.

**FIG. 2.**
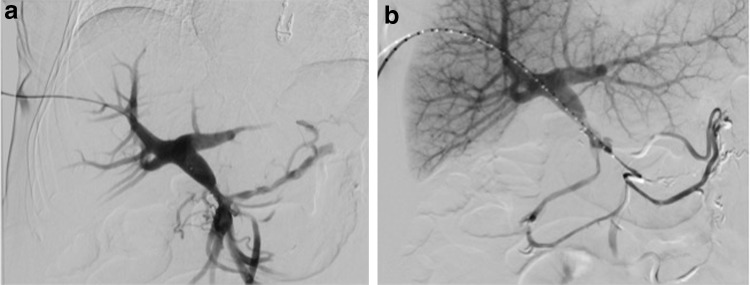
Digital subtraction venograms after right hepatic lobe portal venous system access. **(a)** Early phase venogram shows marked narrowing at the main portal vein–SMV confluence, several small collaterals, and overall slow flow. **(b)** Late phase venogram from a pigtail catheter within the downstream IMV shows continued slow flow with extensive venous collaterals. Faintly visible are previously placed SBRT fiducial markers adjacent (inferior) to the narrowed segment, corresponding to the patient's extrinsic mass. IMV, inferior mesenteric vein; SBRT, stereotactic body radiation therapy.

**FIG. 3.**
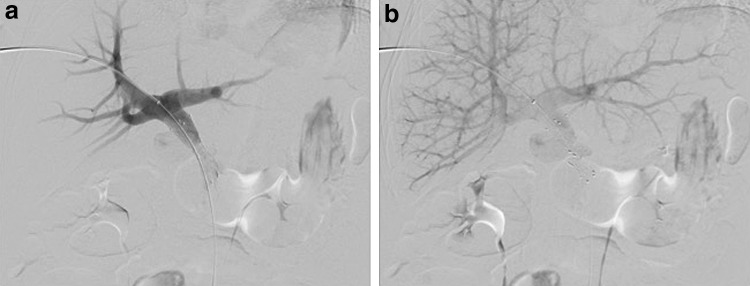
Digital subtraction venograms after portal vein–SMV confluence stent placement across area of narrowing. **(a)** Early phase venogram already shows much improved portal venous flow with stent in place. **(b)** Previously seen extensive collaterals never opacify after stent placement, indicative of improved hemodynamics.

The procedure was performed under general anesthesia. Using direct ultrasound guidance, an AccuStick set (Boston Scientific, Marlborough, MA) was utilized to access a fourth order branch of the portal vein and then exchanged for a 6F 30 cm sheath through an AccuStick dilator. Venography confirmed narrowing of the portal vein–SMV confluence at the level of previously placed radiopaque SBRT fiducial markers (Fig. 2a) and repeat venography from the inferior mesenteric vein demonstrated further significant collateralization (Fig. 2b). Repeat venography from both the SMV and portal vein was performed simultaneously, again demonstrating marked narrowing and collateralization. A 14 × 40 mm self-expanding LifeStar stent (Bard Peripheral Vascular, Inc., Tempe, AZ) was inserted across the area of narrowing, with completion venography demonstrating markedly improved flow with diminished collateralization (Fig. 3a, b). There remained mild flare to the stent because of extrinsic tumor bulk. To prevent irritation of the pancreas, the stent was not initially dilated at the time of placement. The sheath was withdrawn into the hepatic parenchymal tract, and four embolization coils (MWCE-35-14-6-Nester; Cook Medical, Inc., Bloomington, IN) were deployed, with subsequent evidence of hemostasis. The patient was discharged home on the same day, with no postprocedural complications/pain related to the stent placement.

Two weeks later, the patient underwent an uncomplicated open IRE after intraoperative confirmation of nonresectable status because of marked SMV encasement. Treatment was with three sequential ablations in the primary tumor as well as along the hepatic artery (1500 V; 640 total pulses; 70 μs pulse length; 1.5 cm exposure). A gastrojejunostomy for diversion and cholecystectomy was also performed at this time.

Roughly 1 month post-IRE, the patient had elevated LFTs, determined because of a malignant common bile duct stricture requiring endoscopic stent placement, likely exacerbated by postoperative edema. Otherwise, the postoperative course was complicated only by pain, which improved significantly after a successful celiac plexus block.

**FIG. 4.**
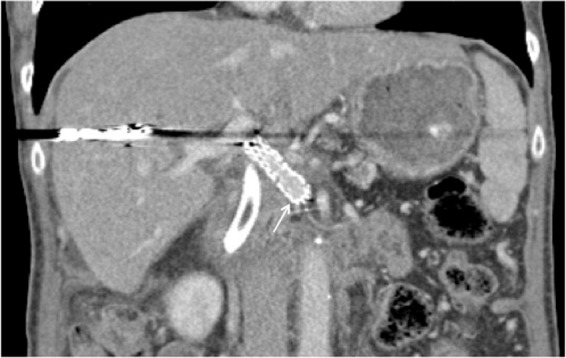
Patent stent at 61 days post-IRE (arrow), with patient treated with aspirin in the interim. Metallic artifact within the liver is because of coils from portal tract access. Postoperative edema of the pancreatic head necessitated endoscopic placement of a CBD stent, also seen. CBD, common bile duct; IRE, irreversible electroporation.

Follow-up CT at 61 days status post-IRE showed a patent portal vein (Fig. 4). The pancreatic head mass was hypoattenuating without significant enhancement or interval enlargement, and the degree of adjacent arterial encasement was not significantly changed. No metastatic disease was present.

## Discussion and Literature Review

Patients with locally advanced unresectable pancreatic head cancer are notoriously difficult to treat. As early evidence shows that IRE could benefit these patients, individual patient selection becomes vitally important for balancing risk versus reward. With many primary pancreatic tumors occurring adjacent to or encasing splanchnic vessels, IRE is promising for its ability to cause nonthermal ablation without vascular damage. Still, potential complications need to be considered.

Most of the published data on IRE for pancreatic tumors and its possible complications are in the form of case reports or case series. A prospective evaluation of 54 patients who underwent IRE for locally advanced pancreatic cancer reported complications of bile leak (two patients), duodenal leak (two patients), and portal vein thrombosis (four patients, including one case resulting in death).^2^ Minor complications reported with IRE include spontaneous pneumothorax related to anesthesia, pancreatitis, bleeding, ascites, and abdominal pain.^2,6^ It should also be noted that patients receiving standard care of chemotherapy and/or radiation are not without complications, commonly including hematological abnormalities, renal failure, liver insufficiency, ascites, nausea, and diarrhea.^2^

Although IRE is appealing because of its nonthermal properties, it has been shown that there can be significant tissue heating and even thermal coagulation with higher voltages and total pulse numbers.^7^ These findings caused early worries about the safety of IRE with metallic stents or clips in or adjacent to the ablation zone. Pertinent to our reported case, this has recently been well studied with both *in vitro* and *in vivo* studies proving IRE causes no heating of the metallic stent itself; in fact tissue in contact with the stent was circumferentially spared (we used the same voltage parameters as in this study).^8^

Although only a single case, it is promising that our patient had no periprocedural complications and was able to be discharged home on the same day. She remains living 24 months after her initial diagnosis.

## Conclusion

This report demonstrates successful use of portomesenteric stenting to reduce postoperative risk of thrombosis, allowing for IRE therapy in a patient without other treatment options. This technique may expand the population eligible for therapy in this clinical circumstance.

## Abbreviations Used

CBD: common bile duct
CT: computed tomography
IMV: inferior mesenteric vein
IRE: irreversible electroporation
LFT: liver function test
SBRT: stereotactic body radiation therapy
SMV: superior mesenteric vein
